# German Health Update: New data for Germany and Europe: The background to and methodology applied in GEDA 2014/2015-EHIS

**DOI:** 10.17886/RKI-GBE-2017-023

**Published:** 2017-03-15

**Authors:** Anke-Christine Saß, Cornelia Lange, Jonas D. Finger, Jennifer Allen, Sabine Born, Jens Hoebel, Ronny Kuhnert, Stephan Müters, Jürgen Thelen, Patrick Schmich, Marike Varga, Elena von der Lippe, Matthias Wetzstein, Thomas Ziese

**Affiliations:** Robert Koch Institute, Department for Epidemiology and Health Monitoring, Berlin, Germany

**Keywords:** STUDY METHODOLOGY, EUROPE, ADULTS, HEALTH SURVEY, HEALTH MONITORING

## Abstract

GEDA 2014/2015-EHIS is an up-to-date health survey of the adult population undertaken within the framework of the Robert Koch Institute’s (RKI) health monitoring system. It uses the EHIS (European Health Interview Survey) Wave 2 questionnaire and includes four modules covering health status, health care, health determinants, and socio-economic variables. Data on nationally relevant issues is also collected. The study employs a mixed-mode design, using both online and paper-based questionnaires to gather data from 24,016 people aged 18 and above: the response rate was 26.9%. The Statistical Office of the European Union (Eurostat) provides prepared data from 28 European Union (EU) member states (plus Norway and Iceland) on the Eurostat website. National analyses for Germany are published as Fact sheets on health reporting in the Journal of Health Monitoring.

## 1. Background

The German Health Update (GEDA) is a nationwide survey of the adult population in Germany conducted by the Robert Koch Institute (RKI) on behalf of the German Federal Ministry of Health. It is conducted within the framework of the RKI’s population-based health monitoring system. The German Health Interview and Examination Survey for Children and Adolescents (KiGGS) [1] and the German Health Interview and Examination Survey for Adults (DEGS) [2] also form part of the RKI’s health monitoring system. In addition to surveys, physical examinations and tests are conducted for KiGGS and DEGS. Health monitoring is aimed at providing reliable information about the population’s health status, health-related behaviour and health care provision. The data gathered for these studies act as the foundation for the German Federal Health Reporting and are used for epidemiological analyses and research into many important public health issues [3].

Three waves of the GEDA study were conducted between 2009 and 2012 as telephone surveys of more than 60,000 respondents [4]. The results have been published as core health indicators [5-7]. The European Health Interview Survey (EHIS Wave 2) was integrated into the GEDA study for the first time as part of GEDA 2014/2015 [8]. Data collection was conducted via a self-administered paper or online questionnaire. The EHIS study is to be harmonised, and the data it gathers will be comparable with that from other European Union (EU) member states (and Norway and Iceland). This enables the EHIS dataset to serve as a basis for national and European health policy and health reporting. Moreover, data from EHIS is made available for epidemiological analyses and international comparative studies, and is integrated into the European Core Health Indicators (ECHI) [9, 10]. 17 EU countries took part in the first wave of EHIS. At that time, the EU member states were under no legal obligation to participate in the study. The second wave of EHIS was undertaken between 2013 and 2015 in all 28 EU member states (as well as in Norway and Iceland) in accordance with a European Commission regulation [11, 12]. The study has to be repeated every five years [13].

**Info box: The ISCED classification system**

ISCED (International Standard Classification of Education) was used to ensure that the data gathered from the participants was comparable [17]. This included condensing the data into three skill levels: low, medium and high levels of vocational and technical training. The use of the ISCED classification system also means that national qualifications are comparable at the international level.

This article provides a brief overview of the methodology applied in GEDA 2014/2015-EHIS. A detailed description of the study’s methodology has been published elsewhere [14]. The results of the core health indicators from GEDA 2014/2015-EHIS are also published in this and upcoming issues of the Journal of Health Monitoring as Fact sheets. These Fact sheets provide data, brief analyses and descriptions of trends in Germany. Data for a specific indicator is arranged according to gender, age and education using the International Standard Classification of Education (ISCED) (see Info box). The Fact sheets published in this issue describe the prevalence of chronic diseases (hypertension, diabetes mellitus, coronary heart disease, stroke and allergies). The Fact sheets published in the next issue will focus on indicators of health- and risk-related behaviour.

## 2. The methods used in GEDA 2014/2015-EHIS

### 2.1 Sample design

**Fig. 1:**
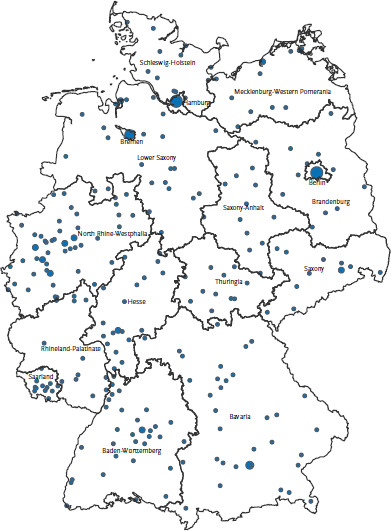
The locations selected for GEDA 2014/2015-EHIS Source: [14]

The GEDA 2014/2015-EHIS study is based on a two-stage stratified cluster sample design. In accordance with EHIS guidelines, the dataset includes the entire population aged 15 and over that has permanent residence in Germany. Analyses for the national GEDA study are based on data from adults aged 18 and over. Sampling was conducted in two phases. The first phase involved the random selection of 301 municipalities aimed at reflecting the different sizes of municipalities and regions in Germany (based on the BIK regional classification system for Germany) [15]. The selection was undertaken by GESIS (the Leibniz Institute for the Social Sciences) in Mannheim, Germany. Selected municipalities with less than 1,000 residents were combined with similarly sized neighbouring communities to form a single locality. In addition, several cities with large populations were represented as a number of communities. The map (see Figure 1) shows the locations that were selected. In the second phase, people with permanent residency in these locations were randomly drawn from local population registers. The required gross sample size for each age group was calculated based on the responses provided to the preliminary tests. The required net sample size was calculated by the Statistical Office of the European Union (Eurostat) for Germany as n=15,260 [8]. However, in order to obtain more precise estimates and to enable regional analyses of each of the 16 German federal states, the sample size was revised upwards to n=20,000 [16]. Consequently, each of the 301 locations required an average of 67 participants.

### 2.2 Questionnaire

The GEDA 2014/2015-EHIS study used a questionnaire; it did not include physical examinations or laboratory tests. The questionnaire for GEDA 2014/2015-EHIS consisted of two components: the questionnaire from EHIS Wave 2 (which was used in all participating countries) and questions that were only asked in Germany. By providing additional questions about specific national issues, it was possible to obtain a time series from GEDA [4] and information about further relevant topics in Germany. The EHIS Wave 2 questionnaire consists of the following four modules:

► health status (self-awareness, chronic diseases, limitations, mental health, pain, accidents, etc.)
► health determinants (smoking, alcohol consumption, weight, physical activity, dietary habits, etc.)
► health care (use of different types of health services such as hospitalisation, outpatient visits, prevention, medication, unmet health care needs)
► background variables on demographic, geographical and socio-economic characteristics of respondents (gender, age, education, household type, etc.).

The questionnaire for EHIS Wave 2 was translated according to the recommended translation protocol [5]. Some modules used validated German versions of the questionnaire. Adjustments were also made to the language deployed in order to meet the requirements of self-administered questionnaires (whether on paper or via the internet). Alongside the national questions, the EHIS Wave 2 questionnaire was expanded to include modules on health literacy, stroke-related knowledge, subjective socio-economic status and working conditions. The questionnaire is published (only in German) as a supplement to the current German issue of the Journal of Health Monitoring. It can be used for research as long as the source is properly cited.

## 3. Distribution, data protection, quality assurance

Data collection for GEDA 2014/2015-EHIS took place between November 2014 and July 2015. The study was approved by the German Federal Commissioner for Data Protection. Participation in the study was voluntary, with the participants informed about the objectives and content of the study as well as data protection. All participants provided their informed consent.

The gross sample amounted to 92,771 people (aged 15 and above). In order to account for seasonal variations, the sample was randomly divided into two tranches. Data collection for the first tranche was conducted in autumn and winter 2014, with data collection for the second tranche occurring in spring and summer 2015. Based on experience gained from various other methodologically-oriented studies, GEDA 2014/2015-EHIS used a sequential mixed-mode design; this was the first time that this had been done for a GEDA study. GEDA 2014/2015-EHIS employed self-administered questionnaires that could be filled out over the internet or on paper [18-20]. All participants initially received a letter inviting them to complete the online questionnaire. This letter contained the relevant URL, the registration code for accessing the online questionnaire, the consent form as well as detailed information about the study and data protection. A reminder was sent out four weeks later. The reminder included a paper questionnaire (as well as a consent form), alongside the URL and registration code. Four weeks after this, a second paper reminder was sent out. A telephone helpline was available during the eleven-month survey period for everyone who had been invited to participate. A study website was also set up to provide information.

Respondents aged between 15 and 34 were offered a €10 voucher to encourage participation. Respondents aged 35 or above had the chance of participating in a lottery in which they could win a €50 voucher. Regional and local newspapers were also contacted, and a press release with important information about the study was sent out with the request for publication.

Extensive quality assurance measures were carried out during the field phase. The data management process for GEDA 2014/2015-EHIS was coordinated by the Epidemiological Data Centre at the Robert Koch Institute. In creating the dataset, the quality and validation guidelines stipulated by Eurostat for EHIS Wave 2 were implemented [8]. The dataset was transmitted to Eurostat in June 2016, which then tested, approved and certified its validity.

## 4. Response rate and net sample

The data analysis for Germany was based on a sample of the participants aged 18 and above. A total of 24,016 fully-completed questionnaires were available from this age group; 10,723 of which had been completed online (44.6%), with 13,293 completed on paper (55.4%). The response rate, which was 26.9% (women 27.5%; men 25.3%), was calculated according to standards developed by the American Association for Public Opinion Research (AAPOR) [21] and reflects the typically low rates gained from other population-based health surveys. The rejection rate was 6.8% (cases where participation in the survey was rejected or discontinued). The highest response rate was observed among the 55 to 74 age group. Among men between 65 and 74, for example, the response rate was 35.2% (women 31.7%); the response rate among men belonging to the youngest age group (18 to 24) was just 22.4% (women 34.6%). Differences were also observed between genders: the response rate was higher for women under 65, and higher for men aged 65 or above. Lange et al. provides detailed information about sampling coverage and the composition of the net sample [14].

## 5. Weighting

A weighting factor was deployed which corrects deviations in the sample from the German population structure. Both design and adjustment weighting were used. Design weighting takes into account the probability of selecting a particular location for study and the probability of selecting a particular participant within a given location. Adjustment weighting enables age and gender within the federal states to be extrapolated to the entire population (as of 31 December 2014) as well as adjustments to be made for location types and regional distribution within Germany. These weighting factors mean that international comparisons can be performed with EHIS data. An additional adjustment was made for national analyses to correct for differences in the educational level of the population in accordance with the guidelines accompanying the 2013 micro-census. This weighting factor is also used in the GEDA Fact sheets that present the results of selected health indicators for Germany (hypertension, diabetes mellitus, coronary heart disease, stroke and allergies).

## 6. Closing remarks

The implementation of the European Health Interview Survey (EHIS) as part of the RKI’s health monitoring system provides numerous opportunities for data analysis and insights at the European level. The Eurostat website provides prevalence data from EHIS Wave 2 (macro-data) for 30 European countries (28 EU member states plus Norway and Iceland) [22] (see also Fehr et al. [23] in this issue). Micro-data from EHIS (individual data from participants from 28 EU member states) should be available for research purposes by the end of 2017 from Eurostat on request.

The variables selected for EHIS Wave 2 were developed through an extensive process leading to consensus between various European countries. However, the final selection posed a methodological challenge for health monitoring in Germany: even when individual variables were supplemented for GEDA 2014/2015-EHIS, current data could not be gathered for all of the indicators that have been studied in Germany in the past. Moreover, despite the modifications made to the study design (online and paper questionnaires were deployed using a sample gained from the registry office instead of using telephone surveys based on a sample of landline telephone numbers), gaps in the time series could not be avoided. Nevertheless, the adjustments made to the GEDA study design mean that it more closely resembles DEGS and KiGGS. These two health monitoring studies, which include physical examinations and tests, also use a written survey among a sample taken from the population register. For a number of years, telephone studies that have not involved prior contact to potential participants have seen a continual reduction in response rates [7]. This problem also affected the GEDA waves conducted between 2009 and 2012. However, changes to the survey mode that were implemented for GEDA 2014/2015-EHIS have been able to halt this trend [14].

## Key statements

- EHIS (European Health Interview Survey) Wave 2 was integrated into the health monitoring conducted by the Robert Koch Institute as part of GEDA 2014/2015-EHIS.
- More than 24,000 adults from Germany participated in the GEDA 2014/2015-EHIS study.
- GEDA 2014/2015-EHIS had a response rate of 26.9%.
- Data for Germany is published in this journal; EHIS data from around 30 European countries is available on the Eurostat website.
